# Effects of blood flow restriction combined with robot-assisted training on upper limb motor function after stroke: a randomized controlled trial protocol

**DOI:** 10.3389/fneur.2026.1807325

**Published:** 2026-04-30

**Authors:** Qian Zhang, Dan Bao, Hewei Wang, Yi Shao, Wenjuan Ding, Li Zhang, Lianxin Song

**Affiliations:** 1 Department of Hand and Foot Microsurgery, Wuxi Huishan District People’s Hospital, Affiliated Huishan Hospital of Xinglin College, Nantong University, Wuxi, Jiangsu, China; 2 Department of Rehabilitation Medicine, Wuxi Central Rehabilitation Hospital, The Affiliated Mental Health Center of Jiangnan University, Wuxi, Jiangsu, China; 3 Department of Rehabilitation Medicine, Huashan Hospital, Fudan University, Shanghai, China

**Keywords:** blood flow restriction, motor function, robot-assisted training, stroke, upper limb

## Abstract

**Background:**

Upper limb motor dysfunction is common after stroke and substantially restricts independence. Robot-assisted training (RAT) and blood flow restriction training (BFRT) have individually demonstrated potential benefits, but evidence regarding their combined application for upper limb rehabilitation after stroke remains limited.

**Objective:**

This study aims to investigate the effectiveness of RAT combined with BFRT in improving upper limb motor function among stroke survivors.

**Methods and analysis:**

This assessor-blinded randomized controlled trial will enroll 100 participants. Participants will be randomly assigned to one of four groups: (A) upper limb RAT combined with BFRT (UL-RAT + BFRT), (B) UL-RAT combined with sham BFRT, (C) BFRT combined with conventional occupational therapy, or (D) conventional occupational therapy alone. All interventions will be delivered once daily, 5 days per week, for 4 weeks. The outcome measures will include upper limb motor function assessed by the Fugl–Meyer Assessment, Upper limb and muscle strength, surface electromyography, Motricity Index–Upper Limb, grip strength, Action Research Arm Test, and Modified Barthel Index. Outcomes will be assessed at baseline and immediately after the intervention. Data will be analyzed according to the intention-to-treat principle, with statistical significance set at 0.05 (two-sided).

**Conclusion:**

This trial will evaluate the feasibility and potential effectiveness of combining BFRT with RAT for post-stroke upper limb rehabilitation.

**Clinical trial registration:**

https://www.chictr.org.cn/, identifier ChiCTR2500096611.

## Introduction

Stroke is a devastating cerebrovascular event and a leading cause of long-term disability in adults worldwide (1). Upper limb motor impairment is highly prevalent and represents a major barrier to functional independence following stroke (2). Effective upper limb performance relies on coordinated shoulder stability, wrist control, and hand dexterity. However, persistent muscle weakness substantially limits motor recovery and reduces quality of life (2, 3). Indeed, upper limb strength has been identified as one of the key predictors of functional outcomes after stroke (4). High-load resistance training [approximately 70% of one—repetition maximum (1RM)] is considered essential to induce significant muscle hypertrophy and strength gains within a relatively short period (5, 6). Nevertheless, stroke survivors frequently present limited cardiopulmonary capacity, abnormal muscle tone, deconditioning, and multiple comorbidities, which hinder their tolerance to high-intensity protocols (7). Therefore, developing low-load yet effective strength training strategies tailored to stroke survivors is of great clinical importance.

Blood flow restriction training (BFRT) is an innovative exercise method that partially restricts arterial inflow and venous return during physical activity by applying pneumatic cuffs proximally on the limbs (8, 9). This technique allows low-load resistance exercises to produce muscle hypertrophy and strength gains comparable to traditional high-load training (10). Although the precise mechanisms remain unclear, metabolic stress caused by hypoxic conditions, accelerated muscular fatigue, and activation of anabolic signaling pathways have been proposed as potential contributing factors (11, 12). BFRT offers advantages such as low cost, short training durations, and high tolerability, making it particularly valuable for individuals with limited exercise capacity (13). Studies involving healthy adults, athletes, and individuals recovering from upper limb injuries have demonstrated improvements in muscle strength, endurance, and morphological adaptations (14, 15). This makes it a promising option for neurorehabilitation.

Despite these advantages, the use of BFRT in stroke rehabilitation remains limited. Current clinical evidence predominantly addresses improvements in lower limb muscle strength and motor function (16–18). For example, Ahmed et al. reported that low-intensity BFRT significantly enhanced lower extremity strength and mobility in ischemic stroke survivors (16). Similarly, Li et al. observed beneficial effects of BFRT on lower limb muscles and motor Function in stroke patients (17). Recent investigations have begun exploring BFRT application for the upper limb after stroke. One randomized controlled trial protocol combining BFRT with muscle energy techniques to manage post-stroke upper limb spasticity has been published, but outcomes are not yet reported (19). To date, studies targeting the upper limb remain limited, and the therapeutic potential of BFRT for upper limb motor recovery after stroke is not fully stablished. Therefore, there is a critical need to identify effective and feasible BFRT-based strategies for upper limb rehabilitation in stroke patients.

Emerging evidence suggests that BFRT may influence motor recovery not only through peripheral muscular adaptations but also via central neurophysiological mechanisms. In patients with stroke, a recent functional near-infrared spectroscopy study has demonstrated that low-load BFRT significantly increases cortical activation in motor-related regions, including the primary motor cortex (M1) and premotor–supplementary motor areas (PMC–SMA) (20). Notably, these neural changes were positively correlated with improvements in motor performance, including Fugl–Meyer scores (20), suggesting a functional link between BFRT-induced cortical activation and motor recovery. Consistent findings from studies further indicate that BFRT can enhance cortical oxygenation, functional connectivity, and afferent feedback in a pressure-dependent manner (21, 22). In addition, BFRT may promote neuroplasticity through metabolic stress-induced upregulation of neurotrophic factors such as brain-derived neurotrophic factor (BDNF) (23). Collectively, these findings support a peripheral-to-central pathway, whereby BFRT may facilitate motor relearning by modulating corticospinal excitability and cortical network activity.

Upper limb motor dysfunction after stroke involves not only muscle weakness but also impaired motor control and coordination deficits. Interventions that can simultaneously enhance neural activation and support task-specific motor relearning may be particularly advantageous. Repetitive, task-specific training is critical for promoting motor learning and facilitating neuroplasticity (24, 25). However, most existing BFRT protocols emphasize lower limb activities such as squatting, resistance exercises, walking, or cycling, which lack sufficient relevance and engagement for patients with upper limb impairments. Robot-assisted training (RAT) provides intensity, repetitive, and task-oriented exercises within enriched, game-based environments, thereby enhancing patient motivation and adherence (26, 27). Additionally, its three-dimensional movement patterns, functional activities of daily living, improving joint coordination and neuromuscular control, and ultimately facilitating upper limb recovery (28, 29). Recent umbrella meta-analysis evidence supports RAT’s effectiveness in enhancing motor function and muscle strength in stroke patients motor function and muscle strength in stroke patients, although these improvements often fall short of clinically meaningful thresholds (30). Whether integrating BFRT with RAT could yield clinically significant enhancements remains unknown.

When integrated with RAT, which provides high-intensity, repetitive, and task-specific motor practice, BFRT-induced neural and neuromuscular adaptations may enhance motor learning processes. Specifically, BFRT may increase the responsiveness of the central nervous system to training stimuli, while RAT delivers structured, goal-directed practice necessary for skill acquisition and functional recovery. Therefore, the combination of BFRT and RAT may produce synergistic effects through the interaction of metabolic stress, neural activation, and task-specific motor learning. To our knowledge, no studies have yet evaluated their combined application for upper limb recovery after stroke. This protocol describes a randomized controlled trial designed to examine the feasibility, safety, and preliminary efficacy of this integrated intervention. We hypothesize that combined intervention will results in greater improvements in upper limb motor performance, muscle strength, and activities of daily living compared with RAT alone.

## Methods

### Study design

**Figure 1:**
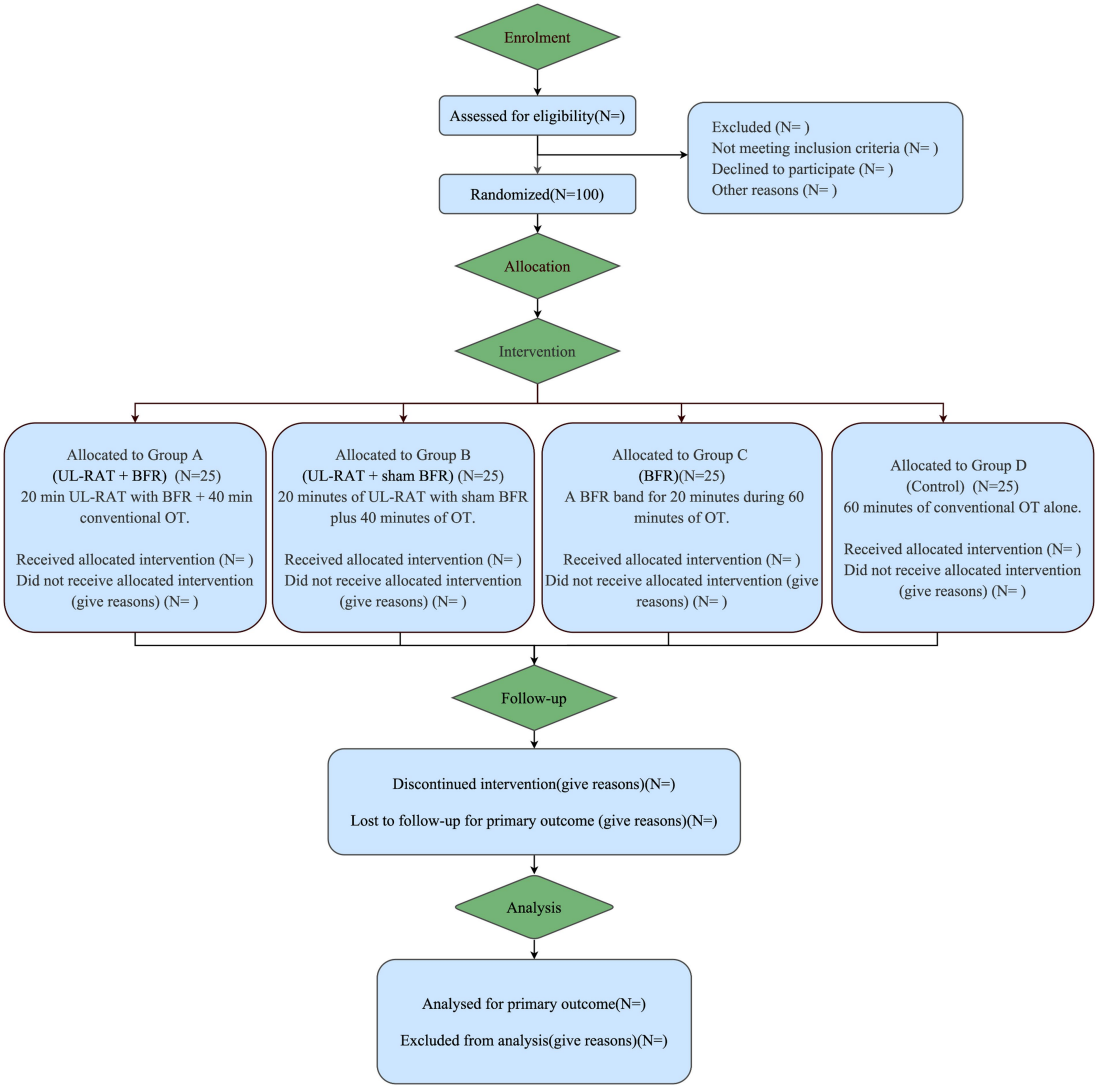
CONSORT diagram. UL-RAT, upper limb—robot assisted therapy; BFR, blood flow restriction.

This single-center, four-arm, parallel-group randomized controlled trial will be conducted according to SPIRIT (Standard Protocol Items: Recommendations for Interventional Trials) guidelines and reported following the CONSORT (Consolidated Standards of Reporting Trials) statement. Outcome assessments will occur at: baseline (pre-intervention), immediately after intervention completion, and at a 4-week follow-up (Figure 1). The trial protocol received ethical approval from the Institutional Review Board of Wuxi Mental Health Center/Wuxi Central Rehabilitation Hospital (Approval No.: WXMHCIRB2024LLky017) and was prospectively registered with the Chinese Clinical Trial Registry (Registration No.: ChiCTR2500096611).

### Participants

Inclusion criteria: (1) diagnosis of stroke confirmed according to the Diagnostic Essentials of Various Cerebrovascular Diseases in China (2019 edition); (2) aged 40–80 years; (3) stroke onset within ≤6 months; (4) first-ever stroke resulting in unilateral hemiplegia; (5) Brunnstrom stage for upper limb >III; (6) Modified Ashworth Scale for upper limb <Grade II; (7) capable of following simple verbal instructions; (8) sitting balance level ≥2; and (9) signed informed consent form. Exclusion criteria: (1) upper limb venous thrombosis detected via ultrasound; (2) severe spasticity or significantly limited range of motion in the affected limb; (3) severe insufficiency of vital organs such as the heart, lungs, liver, or kidneys; (4) severe cognitive or communication impairments; (5) thrombocytopenia or coagulation disorders; (6) absence of skull integrity (e.g., following craniectomy); (7) large-area cerebral infarction or hemorrhage; and (8) current participation in other clinical trials.

### Sample size estimate

Sample size was determined based on the primary comparison between the UL-RAT + BFRT group and the UL-RAT group. A minimal clinically important difference of 9 points on the Fugl-Meyer Assessment of the Upper Limb (FMA-UL) was adopted, which has been estimated for subacute stroke patients using anchor-based methods with the modified Rankin Scale (31). A standard deviation of 10 points was assumed, consistent with previous studies reporting FMA-UE scores in stroke populations (32). Using a two-sample t-test with a two-sided *α* of 0.05 and 80% power, the required sample size was calculated as 20 participants per group (G*Power 3.1.9.7). Although four parallel groups (UL-RAT + BFRT, UL-RAT, BFRT, and control) are included, additional groups primarily serve exploratory purposes. Assuming a 20% dropout rate, the total sample size was increased to 100 participants, resulting in 25 participants in each group.

### Randomization and blinding

An independent researcher not involved in recruitment, intervention delivery, or outcome assessment generated a simple random allocation sequence via computer-based randomization at a 1:1:1:1 ratio. Group assignments were sealed in opaque, sequentially numbered envelopes prepared prior to the study and securely stored by the same researcher. After participants completed baseline assessments and eligibility was confirmed, a research assistant uninvolved in intervention or assessments opened the next sequential envelope privately and disclosed the assigned group on a sealed note to the therapist before the initial intervention session. Outcome assessors and data analysts will remain blinded to group allocation throughout data collection and analysis. Due to the nature of robot-assisted upper limb interventions, blinding of treating therapists is not possible.

### Intervention

**Figure 2:**
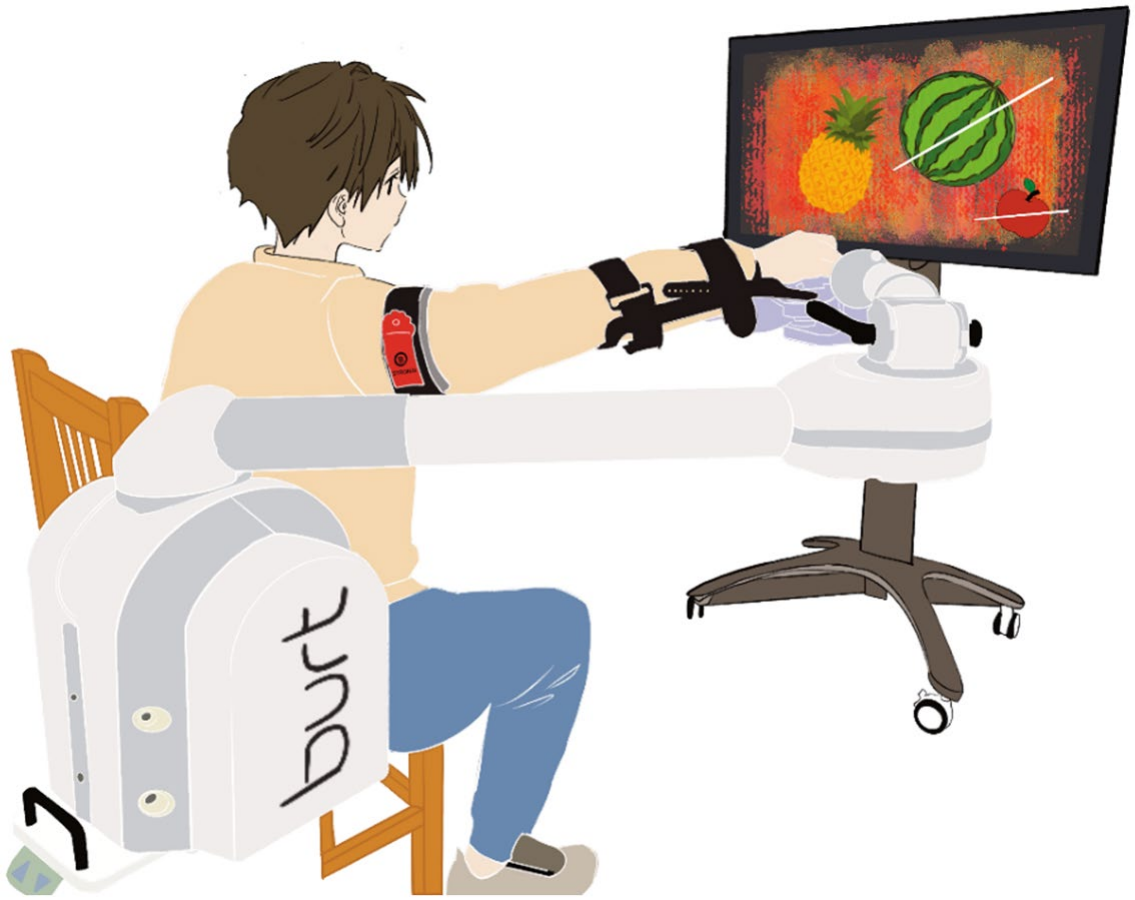
Illustration of robot-assisted training combined with blood flow restriction training.

One hundred participants will be recruited from Wuxi Central Rehabilitation Hospital over 3 years and randomly allocated into four groups. All groups will receive a total of 60 min of training per session to ensure comparable intervention intensity. Conventional occupational therapy will consist of 20 min of therapist-guided training followed by 40 min of supervised self-directed practice. Group A (UL-RAT + BFRT) will receive 20 min of UL-RAT combined with BFRT (Figure 2), followed by 40 min of conventional occupational therapy. Group B (UL-RAT + sham BFRT) will receive 20 min of UL-RAT with sham BFRT and 40 min of occupational therapy. Group C will receive 60 min of occupational therapy, during which BFRT will be applied during the therapist-guided training period. Group D will receive 60 min of conventional occupational therapy alone. All interventions will be administered once daily, 5 days per week, for a total duration of 4 weeks. Perceived exertion will be continuously monitored using the Borg Rating of Perceived Exertion (RPE) scale (6–20 points) (33), which has been shown to correlate well with physiological indicators such as heart rate. Training intensity will be adjusted to maintain RPE within a moderate range (≤13 points) (34).

The upper limb rehabilitation robot (Nanjing ESTUN Company) will be used in this study is a three-dimensional end-effector device designed to facilitate shoulder flexion and elbow flexion-extension movements (35). Before each session, the therapist will adjust the device height to accommodate the participant’s seated position, place the participant’s forearm securely in the designated slot, ensure neutral wrist alignment, and confirm a secure handle grip. All participants will perform active exercises with individualized weight compensation determined at baseline and adjusted as necessary based on performance. Task difficulty and assistance levels will be progressively modified according to predetermined criteria, including task completion rates and movement quality, to ensure appropriate training intensity. Participants will engage in four 5-min game-based tasks selected from standardized interactive options (e.g., fruit fight, airplane fight, basketball shooting, mosquito fighting, and ocean exploration), based on their preferences. The total active training duration will be 20 min.

Blood flow restriction will be applied using a B STRONG BFR training band (Park City, UT, United States) positioned at the proximal third of the upper arm. Before each session, limb occlusion pressure will be measured with a vascular Doppler device (VD-30 series, Hadeco, Japan). Training pressure will be set at 40% of arterial occlusion pressure to ensure participant safety (36), especially considering potential vascular impairment following stroke (37, 38). BFRT will be continuously applied throughout each training session without pressure adjustments between sessions. Participants will be closely monitored throughout each session for adverse signs such as excessive pain, numbness, abnormal skin coloration, or discomfort. Cuffs will be deflated immediately after each session to allow complete reperfusion.

Conventional occupational therapy aims to improve upper limb motor function through a structured and standardized program. Sessions will last 60 min each day, comprising 20 min of individualized therapist-guided training followed by 40 min of supervised self-directed practice. Therapy will include passive and active range-of-motion exercises (e.g., roller and plate exercises), fine motor training (e.g., gripping, releasing, finger opposition), and functional task-oriented training for activities of daily living (e.g., drinking water, grasping small objects, twisting bottle caps, stacking blocks, completing jigsaw puzzles). Progressive resistance training using equipment such as sandbags and dumbbells will be incorporated to enhance upper limb strength. All occupational therapy sessions will adhere to a standardized protocol. Although individual adjustments based on each participant’s functional status will be permitted, the overall structure, duration, and therapeutic components will remain consistent. Therapists will receive training prior to the study to ensure uniform intervention delivery.

### Outcomes

The primary outcome will be the FMA-UL motor assessment, a validated and reliable scale for evaluating upper limb motor function in patients during the subacute stroke phase (39). Only the upper limb section will be assessed, with scores ranging from 0 to 66; lower scores represent greater impairment.

Upper limb muscle strength will be assessed using a MicroFET2 handheld dynamometer (Hoggan Industries, Inc., West Jordan, UT, United States) (40). Maximum isometric strength will be measured in 13 muscle groups: anterior, middle, and posterior deltoids; latissimus dorsi; subscapularis; infraspinatus and teres minor; pectoralis major; middle trapezius; rhomboids; triceps; biceps and brachialis; and brachioradialis extensor carpi radialis longus. Each assessment will begin with an explanation and demonstration of movements, safety instructions, and testing procedures, followed by a brief warm-up to prevent muscle strain or fatigue. Strength will be tested using three maximum voluntary contractions per muscle group on both sides, with the average of the three trials recorded as the final value. Measurements will begin with the affected side, followed by at least a 30-s rest before assessing the unaffected side. Participants will be instructed to sustain each contraction for 5 s, aiming for maximum force within the first 3 s.

Surface electromyography (sEMG) will be recorded using a wireless BTS FREEEMG 300 system (41) (BTS Bioengineering, Milan, Italy). Prior to electrode placement, the skin will be prepared by shaving (if necessary) and cleaned with alcohol to reduce impedance. A pair of silver-silver chloride electrodes will be placed over the muscle belly, aligned with muscle fibers and spaced 2 cm apart. The muscles assessed will include the biceps brachii, triceps brachii, deltoid, and wrist extensor muscles. Participants will perform maximal voluntary isometric contractions lasting 7 s each, repeated three times per muscle. The sEMG signals will be sampled at 1,000 Hz and band-pass filtered between 20 and 500 Hz. Data will be recorded for offline analysis using BTS EMG-Analyzer software.

Grip strength will be assessed using a Jamar handheld grip dynamometer (United States) (42). Participants will sit with elbows flexed at 90 degrees and upper arms held close to the body. They will perform maximal isometric contractions by squeezing the dynamometer for at least 5 s. Each hand will be tested three times, with a one-minute rest interval between trials. The highest recorded value will be reported as the final result.

The Motricity Index (MI), which evaluates muscle strength in stroke patients, will also be used (43). The upper limb section includes muscle strength grading for three movements: pinching, elbow flexion, and shoulder abduction. The total MI score, ranging from 0 to 100, is calculated as the sum of the three movement scores plus one; higher scores indicate better muscle strength.

The Action Research Arm Test (ARAT) will evaluate upper limb dysfunction following stroke (44). It consists of 19 items across four movement categories: grasping, gripping, pinching, and gross movements. Performance will be rated on a 4-point scale (0–3), yielding a total possible score of 0–57; higher scores indicate better upper limb function.

The MBI will measure participants’ abilities in activities of daily living (45). It consists of 10 items, with total scores ranging from 0 to 100; higher scores represent greater functional independence.

### Statistical analysis

Statistical analyses will be performed using SPSS version 22.0 (IBM Corp., Armonk, NY, United States). All analyses will follow the intention-to-treat principle. Baseline characteristics will be summarized using means ± standard deviations or median (interquartile ranges) for continuous variables, and frequencies (percentages) for categorical variables. Baseline group differences will be assessed using one-way analysis of variance, Kruskal–Wallis tests, or chi-square tests, as appropriate. A two-factor repeated-measures analysis of variance (group × time) will examine changes over time and group differences, provided assumptions are met. If normality or sphericity assumptions are violated, linear mixed-effects models will be applied. The primary analysis will focus on the between-group difference in change from baseline between the UL-RAT + BFR group and the UL-RAT group. Comparisons involving BFR and control groups will be exploratory. *Post hoc* pairwise comparisons with appropriate adjustments will be conducted when significant interactions or main effects are identified. A two-sided *p*-value < 0.05 will indicate statistical significance.

### Data management and monitoring

Trained research staff, blinded to group allocation, will collect all data. Two independent researchers will enter data using EpiData version 3.1. Discrepancies identified through double-entry comparison will be resolved by reviewing original source documents. Regular checks will ensure data completeness and consistency, and only verified datasets will be used for statistical analyses. All study data will be securely stored with access limited to authorized personnel to maintain confidentiality. Additionally, an independent safety monitor will oversee the study and systematically review adverse events throughout the trial.

### Safety monitoring

BFRT is generally considered safe; however, adverse events such as pain, muscle fatigue, dizziness, and fluctuations in blood pressure have been reported (46, 47). To ensure participant safety, a comprehensive pre-intervention assessment will identify contraindications, including histories of deep vein thrombosis, severe cardiovascular disease, uncontrolled hypertension, or peripheral vascular disorders. During each intervention session, therapists will carefully control cuff pressure and positioning. Cardiovascular parameters, including heart rate, blood pressure, and oxygen saturation, will be monitored before, during, and after each session. Thrombotic risks will be monitored throughout the study by observing clinical signs such as limb swelling, pain, or changes in skin coloration. Participants will be instructed to promptly report any unusual symptoms, and adverse events or feedback will be systematically recorded. Training sessions will immediately terminate if any of the following conditions occur: systolic blood pressure exceeding 180 mmHg or diastolic blood pressure above 110 mmHg; abnormal heart rate or arrhythmia; signs of limb ischemia or thrombosis; severe pain or discomfort; or participant request to discontinue.

### Participant timeline

**Figure 3:**
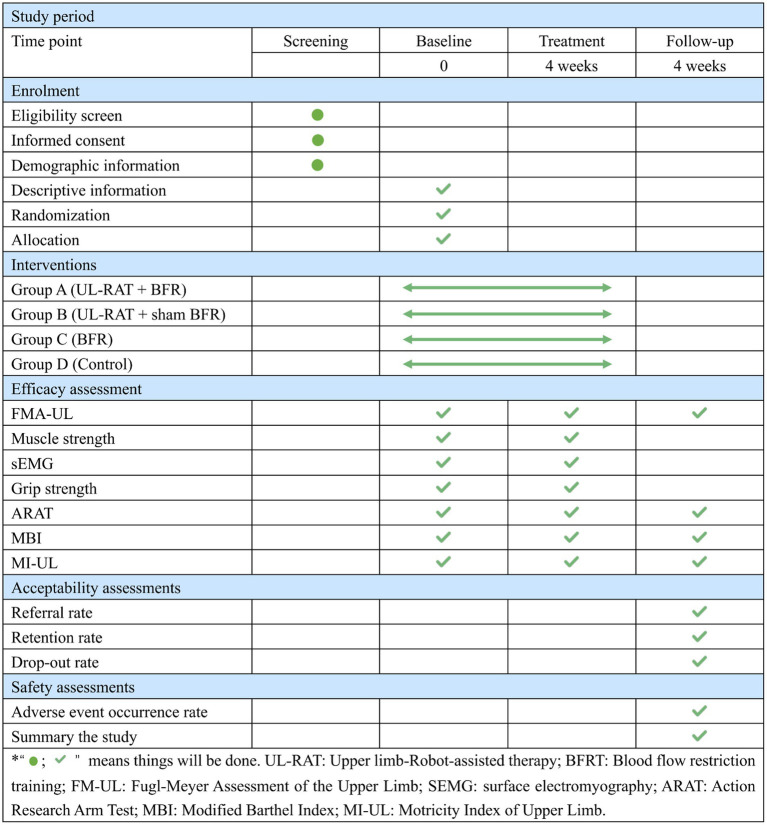
Schedule of enrollment, intervention and assessment.

The schedule of enrollment, interventions, and assessments is presented in Figure 3. The FMA-UL, MI-UL, ARAT, and MBI will be assessed at three time points: baseline (pre-intervention), immediately after intervention completion, and at a 4-week follow-up. Muscle strength, surface electromyography, and grip strength will be assessed only at baseline and immediately post-intervention. In addition, pre-enrollment screening procedures (eligibility confirmation, informed consent) occur before baseline and are not part of the formal outcome assessment.

## Discussion

RAT and BFRT each have demonstrated potential benefits for post-stroke rehabilitation (48, 49). However, evidence regarding their combined application for upper limb recovery remains limited. To date, no randomized controlled trial has assessed the efficacy of combining BFRT with upper limb RAT for improving motor function and muscle strength after stroke. This randomized controlled study aims to evaluate the feasibility and potential effectiveness of this integrated approach through clinical assessments and objective instrumental measurements.

RAT is characterized by high repetition and task-oriented practice (50, 51). Incorporating gamified scenarios and real-time audiovisual feedback helps optimize movement patterns and enhances patient motivation and engagement. Moreover, task-specific training paired with immediate performance feedback may facilitate neuroplasticity and promote conditions favorable for motor learning (52, 53). BFRT employs a different mechanism, inducing metabolic stress and increasing muscle activation at low-intensity loads (54). This method is advantageous for individuals unable to tolerate conventional resistance training. Therefore, integrating BFRT with RAT may offer complementary benefits, addressing both central motor control and peripheral metabolic adaptations, to target upper limb impairment after stroke effectively.

Compared with traditional BFRT protocols, this combined approach enables standardized, functionally relevant repetitive upper limb movements. Robot-assisted systems ensure movement accuracy and controlled training dosage, potentially improving treatment adherence and safety. In addition to clinical scales like the FMA-UL, this study incorporates muscle strength assessments and sEMG measurements. Combining clinical outcomes and objective physiological assessments may enhance interpretation of treatment effects and offer insights into underlying recovery mechanisms. Although BFRT is generally safe, applying it in stroke patients requires careful monitoring due to potential vascular risks. Therefore, this study has implemented a structured safety monitoring and risk mitigation plan to ensure controlled and safe delivery of the intervention.

Several limitations must be acknowledged, including the single-center design, absence of neuroimaging outcomes, and challenges in maintaining blinding of participants and therapists. Additionally, the 4-weeks follow-up period allows evaluation of short-term effects but does not inform long-term outcomes. Future research with longer follow-up durations is necessary to assess the durability of intervention effects. Despite these limitations, this trial will provide preliminary evidence regarding the feasibility of integrating BFRT into upper limb rehabilitation after stroke. The findings will help inform the design of future large-scale trials.

## Conclusion

Combining BFRT with upper limb RAT represents a potentially promising strategy for improving muscle strength and functional performance in stroke patients.
